# Supplementary material for: Modulating gene regulation function by chemically controlled transcription factor clustering
Source: Nat Commun. 2022 May 13;13:2663. doi: 10.1038/s41467-022-30397-2 (PMC9106659; doi:10.1038/s41467-022-30397-2)
Supplement: Supplementary file 1 — Supplementary Information [file 41467_2022_30397_MOESM1_ESM.pdf]

Supplementary Information for

Modulating gene regulation function by chemically controlled transcription factor clustering

Jiegen Wu,[1,2,3,4,#] Baoqiang Chen,[4,#] Yadi Liu,[1,2,3] Liang Ma,[1,2,3] Wen Huang,[1,2,3] and Yihan Lin[1,2,3,*]

# Equal contribution
*Correspondence: yihan.lin@pku.edu.cn

This file contains

Supplementary Figures 1-9

Supplementary Table 1

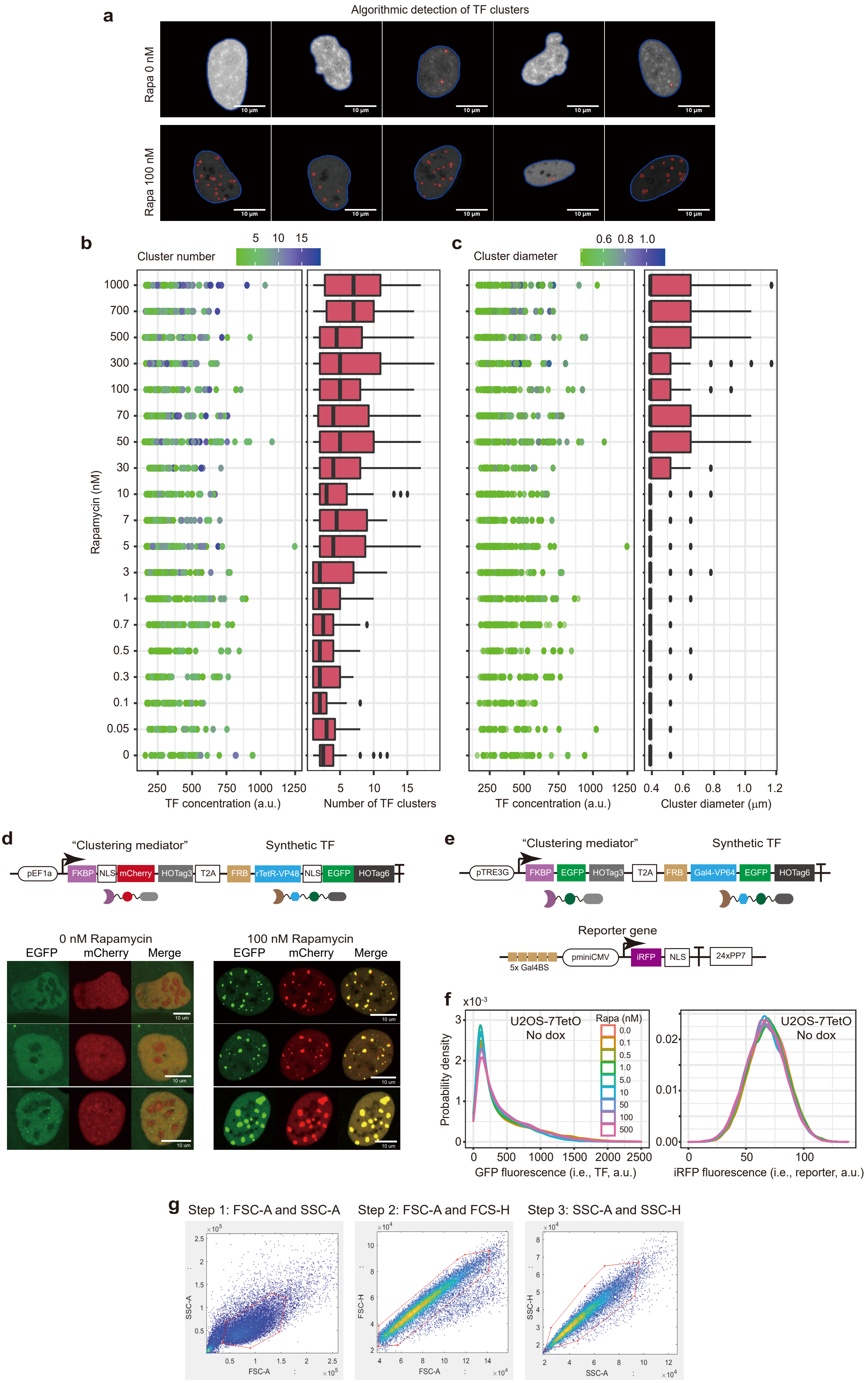

a
Algorithmic detection of TF clusters
Rapa 0 nM
Rapa 100 nM
10 μm
b
Cluster number
5 10 15
Rapamycin (nM)
1000
700
500
300
100
70
50
30
10
7
5
3
1
0.7
0.5
0.3
0.1
0.05
0
250 500 750 1000 1250
TF concentration (a.u.)
5 10 15
Number of TF clusters
c
Cluster diameter
0.6 0.8 1.0
250 500 750 1000 1250
TF concentration (a.u.)
0.4 0.6 0.8 1.0 1.2
Cluster diameter (μm)
d
"Clustering mediator"
Synthetic TF
pEF1a
FKBP
NLS
mCherry
HOTag3
T2A
FRB
rTetR-VP48
NLS
EGFP
HOTag6
0 nM Rapamycin
100 nM Rapamycin
EGFP
mCherry
Merge
10 um
e
"Clustering mediator"
Synthetic TF
pTRE3G
FKBP
EGFP
HOTag3
T2A
FRB
Gal4-VP64
EGFP
HOTag6
Reporter gene
5x Gal4BS
pminiCMV
iRFP
NLS
24xPP7
f
x10-3
U2OS-7TetO
No dox
Rapa (nM)
0.0
0.1
0.5
1.0
5.0
10
50
100
500
Probability density
0 500 1000 1500 2000 2500
GFP fluorescence (i.e., TF, a.u.)
0.00 0.01 0.02
0 50 100
iRFP fluorescence (i.e., reporter, a.u.)
g
Step 1: FSC-A and SSC-A
Step 2: FSC-A and FCS-H
Step 3: SSC-A and SSC-H
SSC-A
FSC-A
FSC-H
SSC-H

Supplementary Figure 1: Additional characterizations of rapamycin-controlled TF clustering.
a, Representative confocal images of U2OS-7TetO cells under 0 nM or 100 nM rapamycin. Each cell image was overlaid with software-detected nucleus contour (blue) and detected TF clusters (red). Images were generated from maximum projection of confocal z-stack. **b-c**, Dot plots and boxplots showing quantifications of TF clustering. Detected numbers of TF clusters and the corresponding TF concentrations (quantified as mean nuclear EGFP intensities) in individual cells (with each dot representing one cell) are plotted for U2OS-7TetO cells under various indicated rapamycin concentrations (**b**). Mean cluster numbers are plotted in Fig. 1e. Analogous plots for mean cluster diameter in individual cells are shown (**c**). Population-averaged cluster diameters are plotted in Fig. 1f. The cell numbers are between 70 and 117 (see source data for the exact number). For boxplots, middle line indicates median and box ranges from first to third quartile, with whiskers extending up to 1.5× interquartile range. **d,** Two-color construct for analyzing interactions between clustering mediator and the synthetic TF. Compared to the U2OS-7TetO system in Fig. 1a, in this two-color construct each component was fused with distinct fluorescent proteins (top). Three representative cells were shown for 0 nM and 100 nM rapamycin conditions separately. Images were generated from maximum projection of confocal z-stack. From these results, it is evident that the formation of TF clusters is controlled by rapamycin-dependent mechanism. **e,** Schematic of the CHO-Gal4 system. The clustering mechanism is the same as the U2OS-7TetO system (i.e., Fig. 1a). It should be noted that, in contrast to the U2OS-7TetO system where doxycycline is used for enabling the DNA binding capability of rTetR, here doxycycline is used to induce the expression of synthetic TF Gal4-VP64 and clustering mediator. **f**, Control experiments showing that rapamycin addition does not affect TF fluorescence (left) and the activation of downstream genes requires the DNA binding capability of the TF (right). **g**, FACS data gating strategy. Note that we only gated for singlets. Source data are provided.

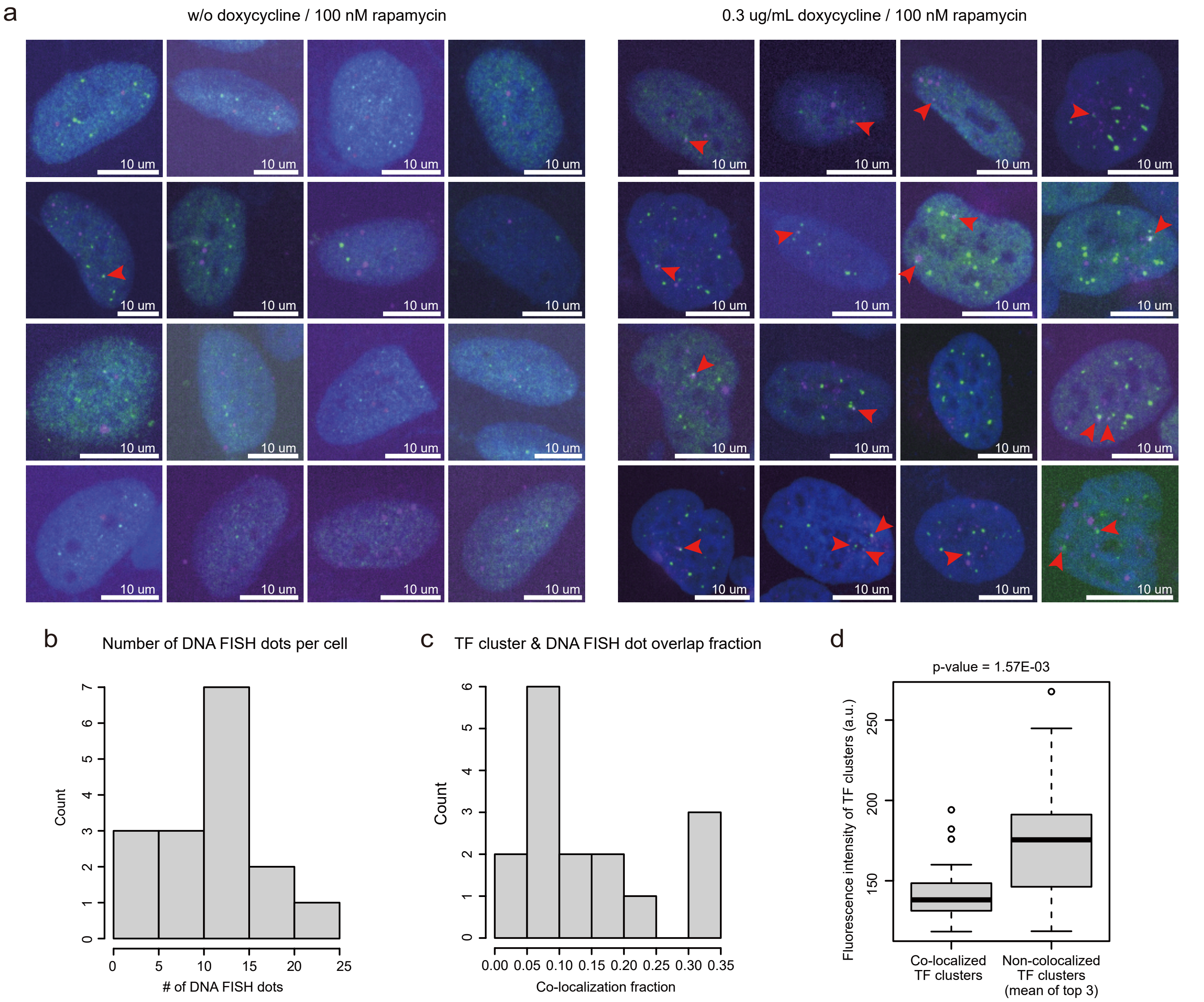

Supplementary Figure 2: Evidence for doxycycline-dependent binding of TF clusters to reporter DNA loci.

a, Three-color merged images of U2OS-7TetO cells under indicated conditions. Green: TF clusters. Purple: DNA fluorescence in situ hybridization (FISH) with probes targeting the reporter gene loci. Blue: DAPI staining of DNA. See Fig. 3b for examples of cells with images of separate colors. For each color, images were generated from maximum projection of confocal z-stack (Methods). Red arrow indicates overlapping TF clusters and DNA FISH signals. **b**, Histogram of the number of detected DNA FISH foci per cell. n = 16 cells. Mean number of dots per cell is 11.4. **c**, Histogram of the fraction of FISH foci co-localizing with TF clusters per cell. n = 16 cells. Mean overlap fraction is 14.7%. **d**, Boxplot comparing the intensity of TF clusters co-localizing versus non-colocalizing with DNA FISH foci in the prescence of doxycycline. The intensity of co-localized TF clusters or the mean intensity of the top 3 largest non-colocalizing TF clusters in the corresponding cell was used. 20 co-localized TF clusters from 16 cells were included in the plot. p value was calculated by paired two-sided t-test ($t = -3.6846$, $df = 19$). For boxplots, horizontal line indicates median and box ranges from first to third quartile, with whiskers extending up to 1.5×interquartile range. Source data are provided.

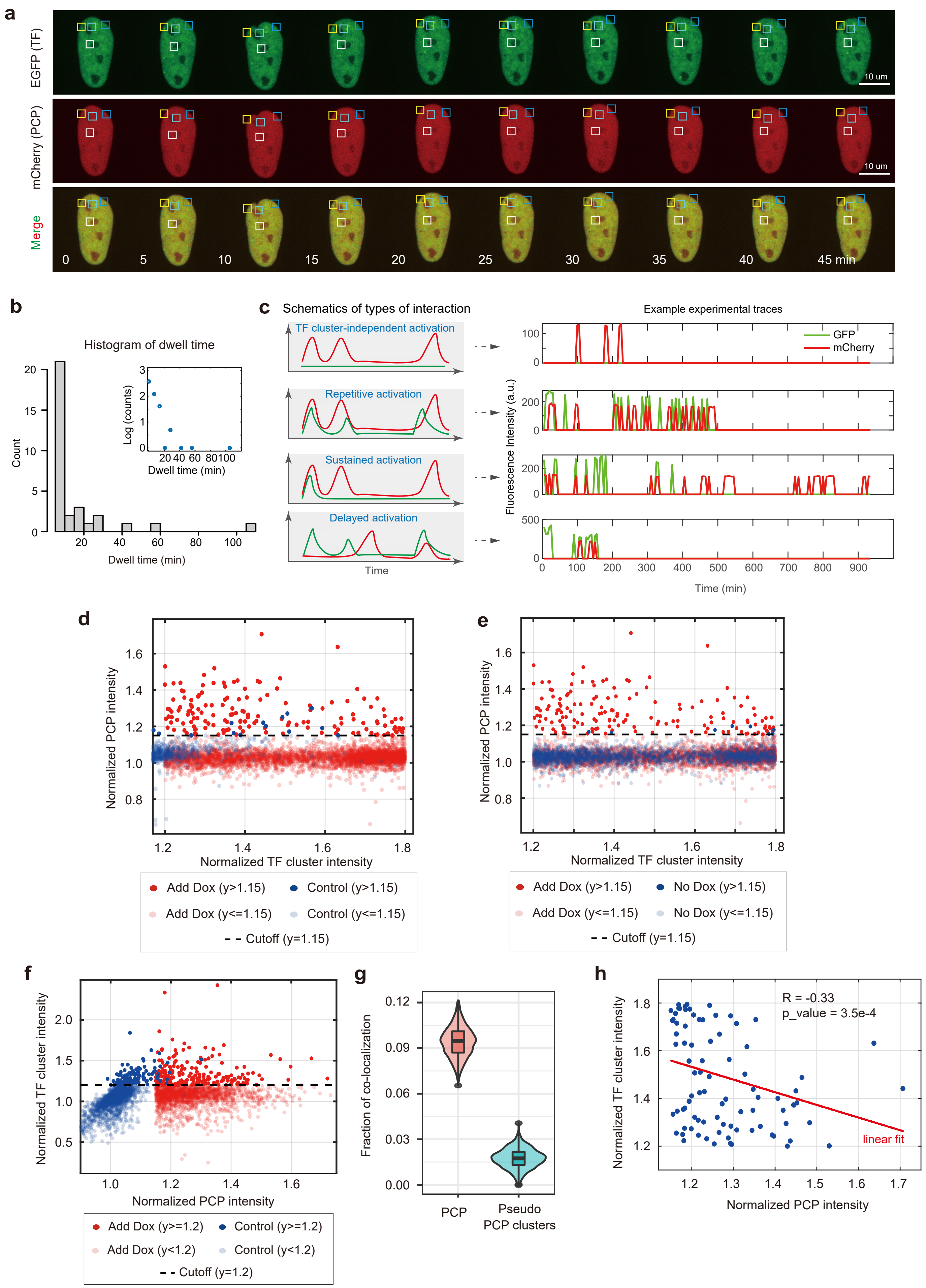

a
EGFP (TF)
mCherry (PCP)
Merge
10 um
0
5
10
15
20
25
30
35
40
45 min
b
Histogram of dwell time
Count
Dwell time (min)
Log (counts)
c
Schematics of types of interaction
TF cluster-independent activation
Repetitive activation
Sustained activation
Delayed activation
Time
Example experimental traces
GFP
mCherry
Fluorescence Intensity (a.u.)
Time (min)
d
Normalized PCP intensity
Normalized TF cluster intensity
Add Dox (y>1.15)
Control (y>1.15)
Add Dox (y<=1.15)
Control (y<=1.15)
Cutoff (y=1.15)
e
Normalized PCP intensity
Normalized TF cluster intensity
Add Dox (y>1.15)
No Dox (y>1.15)
Add Dox (y<=1.15)
No Dox (y<=1.15)
Cutoff (y=1.15)
f
Normalized TF cluster intensity
Normalized PCP intensity
Add Dox (y>=1.2)
Control (y>=1.2)
Add Dox (y<1.2)
Control (y<1.2)
Cutoff (y=1.2)
g
Fraction of co-localization
PCP
Pseudo
PCP clusters
h
R = -0.33
p_value = 3.5e-4
linear fit
Normalized TF cluster intensity
Normalized PCP intensity

Supplementary Figure 3: Evidence supporting that TF clusters can activate the transcription of genome-integrated reporter genes.

a-c, Additional characterizations of spatiotemporal interactions between TF clusters and nascent transcription sites. Time-lapse two-color snapshots of an example cell showing spatiotemporal interactions between TF clusters and four box-highlighted nascent transcription sites of reporter genes (**a**). Note that zoom-in views of the upper right corner of the cell were shown in Fig. 3d. From two-color traces, the dwell time of TF clusters binding to reporter locus could be estimated (**b**, n = 32 events). Insert in **b** displays the histogram in log-linear scale to illustrate the exponential-like behavior. Schematics and example experimental traces of different modes of interaction between TF clusters and nascent transcription sites were shown in **c**. Each pair of experimental traces (GFP and mCherry) represents one reporter locus. Note that classification of interaction mode was purely empirical and may not represent the actual mode of interaction, because imaging was not continuous. See also Supplementary Movie 1. **d-e**, Quantifications of co-localization between TF cluster signals and nascent transcription signals. TF clusters were first identified in EGFP channel and their intensities were normalized by mean cellular EGFP intensity (Methods). The corresponding PCP intensities were calculated in mCherry channel and were normalized by mean cellular mCherry intensity. To control for by-chance co-localization, pseudo-TF clusters were generated as control (Methods). Data for the condition with doxycycline (0.3 µg/mL) are shown together with control data (**d**) or the condition without dox (**e**). Data points above threshold were identified as co-localization. The bootstrapped means of co-localization fractions are shown in Fig. 3g. **f-g**, Analogous quantifications of co-localization by using the number of PCP foci as denominator (n = 615 cells). For boxplots, horizontal line indicates median and box ranges from first to third quartile, with whiskers extending up to 1.5 × interquartile range. **h**, For co-localized foci in the dox condition, PCP signal intensity and TF cluster intensity displayed an appearent negative correlation. Pearson correlation coefficient and p-value were extracted from corr function in Matlab (t = -3.685, df = 112). Source data are provided.

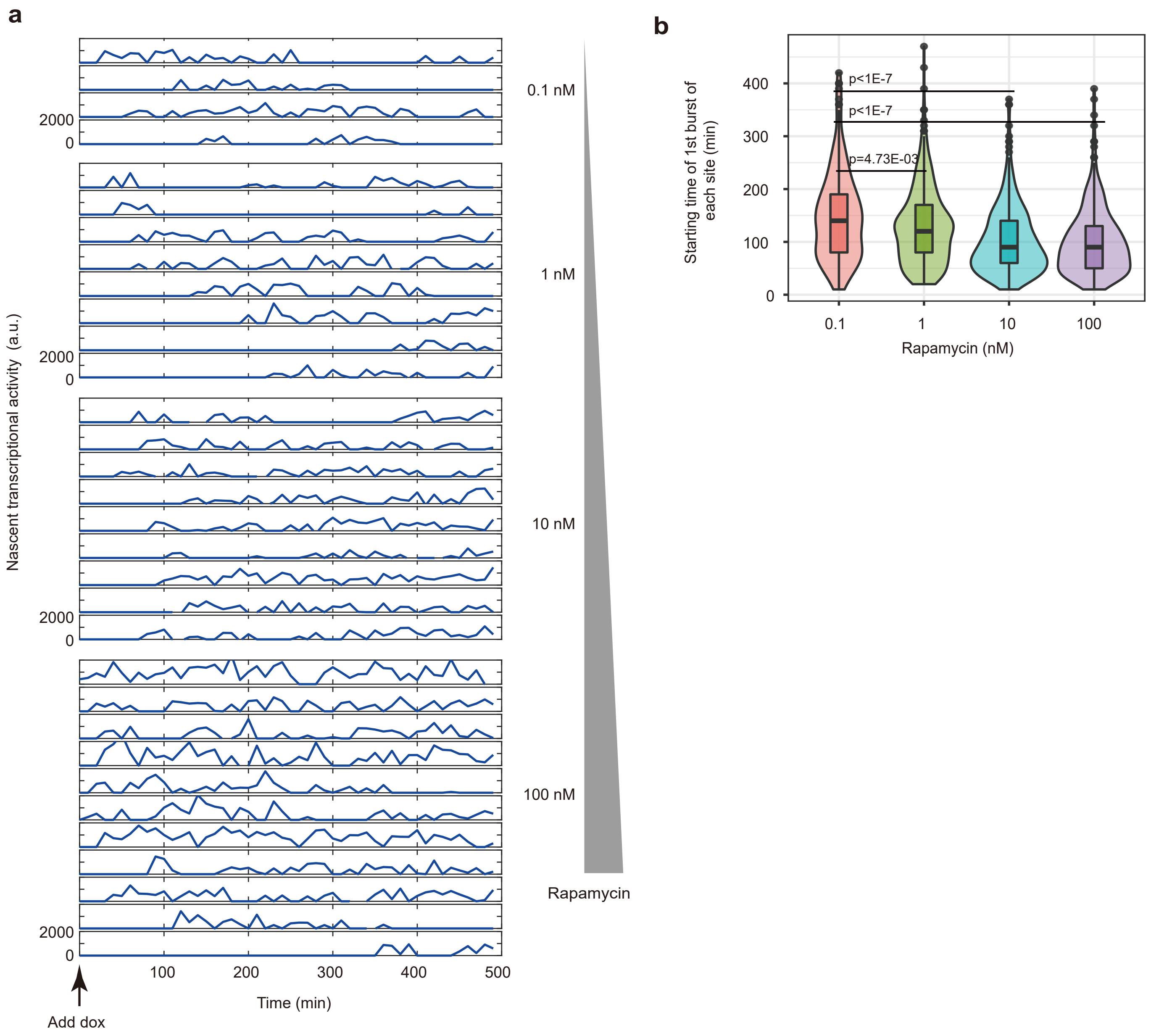

Supplementary Figure 4: Characterization of transcriptional dynamics modulation by TF clustering.

a, Example trajectories showing the nascent transcriptional activities for all detected reporter's nascent sites inside single cells under indicated rapamycin concentrations. It is noted that the number of detected nascent sites increases from top to bottom. See also Supplementary Movie S2. **b**, Time to the first transcriptional burst post-doxycycline addition for each nascent site in cells under indicated rapamycin concentrations. Cell numbers are 36, 46, 40, and 37 from left to right. p values were calculated by one-way ANOVA and TukeyHSD. For boxplots, horizontal line indicates median and box ranges from first to third quartile, with whiskers extending up to 1.5 × interquartile range. Source data are provided, which contain details of statistical tests.

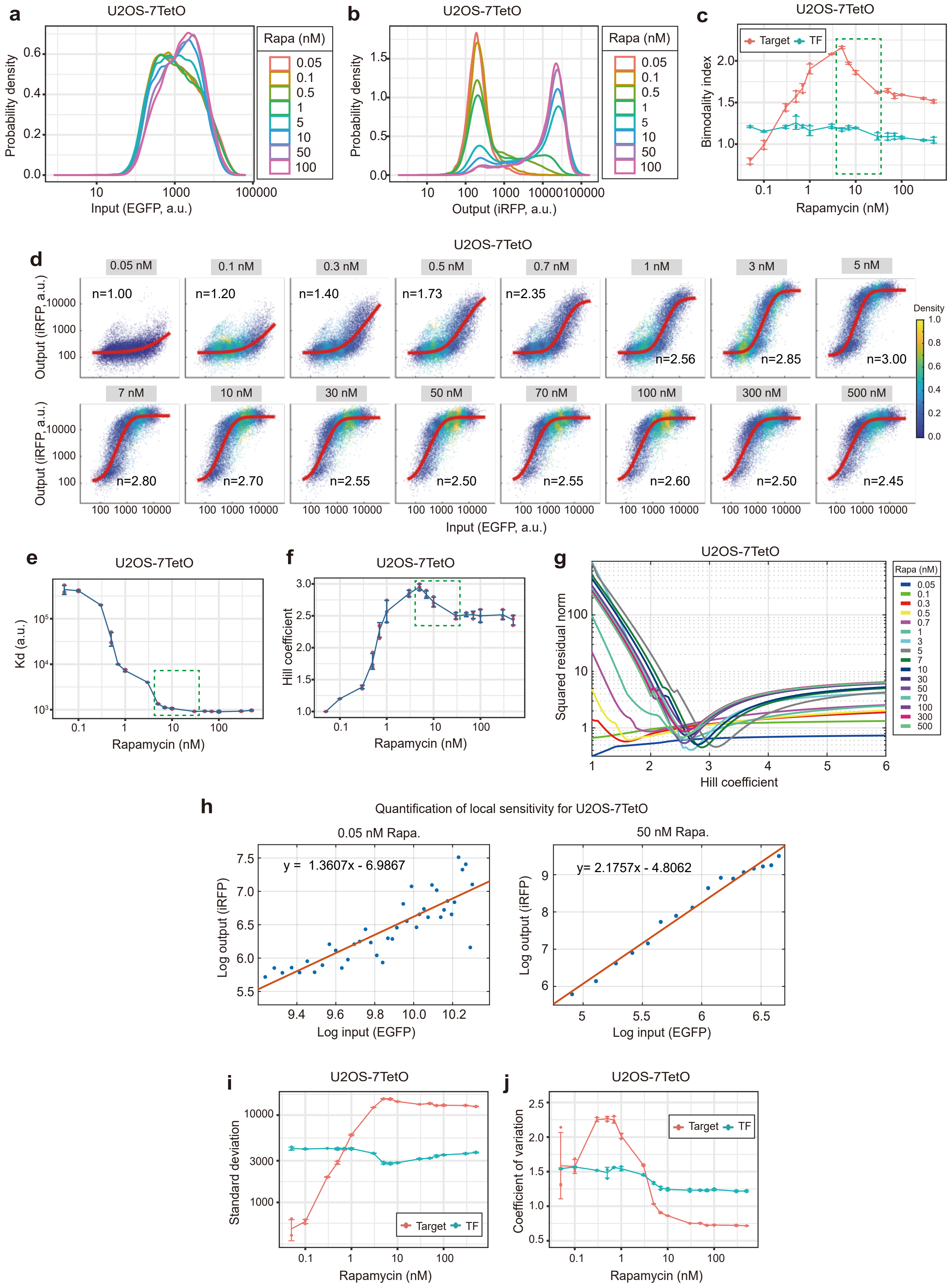
a
U2OS-7TetO
Probability density
Input (EGFP, a.u.)
Rapa (nM)
0.05
0.1
0.5
1
5
10
50
100
b
U2OS-7TetO
Probability density
Output (iRFP, a.u.)
Rapa (nM)
c
U2OS-7TetO
Target
TF
Bimodality index
Rapamycin (nM)
d
U2OS-7TetO
0.05 nM
0.1 nM
0.3 nM
0.5 nM
0.7 nM
1 nM
3 nM
5 nM
n=1.00
n=1.20
n=1.40
n=1.73
n=2.35
n=2.56
n=2.85
n=3.00
7 nM
10 nM
30 nM
50 nM
70 nM
100 nM
300 nM
500 nM
n=2.80
n=2.70
n=2.55
n=2.50
n=2.55
n=2.60
n=2.50
n=2.45
Output (iRFP, a.u.)
Input (EGFP, a.u.)
Density
e
U2OS-7TetO
Kd (a.u.)
Rapamycin (nM)
f
U2OS-7TetO
Hill coefficient
Rapamycin (nM)
g
U2OS-7TetO
Squared residual norm
Hill coefficient
Rapa (nM)
0.05
0.1
0.3
0.5
0.7
1
3
5
7
10
30
50
70
100
300
500
h
Quantification of local sensitivity for U2OS-7TetO
0.05 nM Rapa.
y = 1.3607x - 6.9867
Log output (iRFP)
Log input (EGFP)
50 nM Rapa.
y= 2.1757x - 4.8062
Log output (iRFP)
Log input (EGFP)
i
U2OS-7TetO
Standard deviation
Target
TF
Rapamycin (nM)
j
U2OS-7TetO
Coefficient of variation
Target
TF
Rapamycin (nM)

Supplementary Figure 5: Additional characterizations of the roles of TF clustering in the U2OS-7TetO system.

a-b, Distributions of input (**a**) and output (**b**) signals under indicated rapamycin concentrations for the U2OS-7TetO system. The plots relate to Fig. 4b. **c**, Bimodality indexes of the input and output distributions at indicated rapamycin concentrations. Green boxed region indicates 5 nM to 30 nM rapamycin conditions. Data are presented as mean ± S.D. (n = 3 biological replicates). **d**, Steady-state TF (EGFP) and reporter (iRFP) signals were quantified by flow cytometry at indicated rapamycin concentrations (with 0.3 μg/ml doxycycline) and were fitted by Hill function (red curve). Note that some of the plots were shown in Fig. 4b. **e-f**, The modulation of dissociation constant (**e**) and ultrasensitivity (quantified by Hill coefficient, **e**) by TF clustering propensity. Both parameters were obtained from the fitted Hill functions. Green boxed regions indicate the same range as in panel c. Data are presented as mean ± S.D. (n = 3 biological replicates). **g**, Squared norm of the residual versus Hill coefficient for nonlinear curve fitting of the input-output data at indicated rapamycin concentrations. **h**, Quantification of local sensitivity in the dose resposne curve. Linear region (in log-log scale) of the dose response data of indicated rapamycin concentration was used for fitting. Data were binned before fitting to avoid sampling bias (Methods). **i-j**. Characteristics of the input and output distributions at indicated rapamycin concentrations, including standard deviation (**i**) and coefficient of variation (**j**). Data are presented as mean ± S.D. (n = 3 biological replicates). Source data are provided.

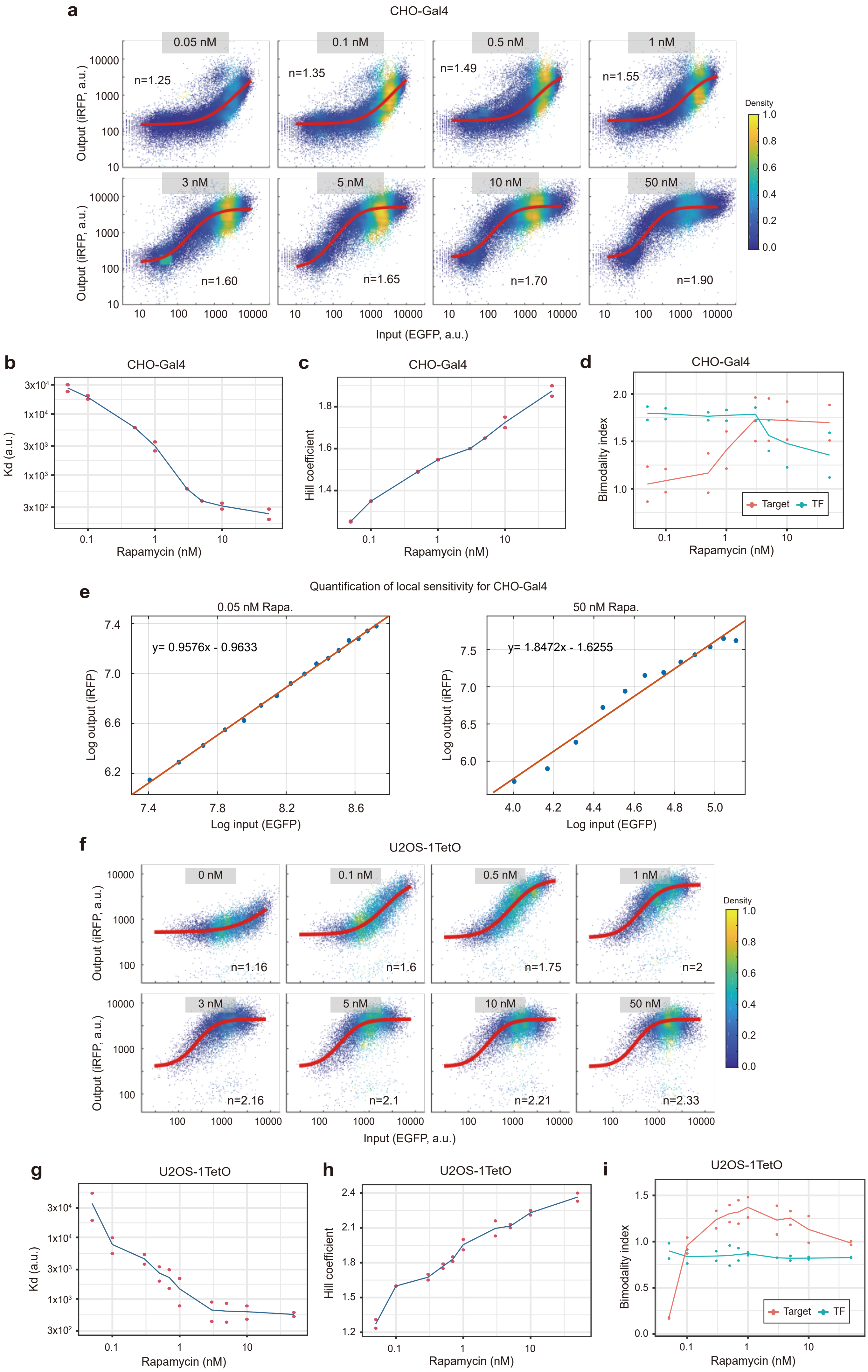
a
CHO-Gal4
0.05 nM
0.1 nM
0.5 nM
1 nM
n=1.25
n=1.35
n=1.49
n=1.55
3 nM
5 nM
10 nM
50 nM
n=1.60
n=1.65
n=1.70
n=1.90
Output (iRFP, a.u.)
Input (EGFP, a.u.)
Density
b
CHO-Gal4
Kd (a.u.)
Rapamycin (nM)
c
CHO-Gal4
Hill coefficient
Rapamycin (nM)
d
CHO-Gal4
Bimodality index
Target
TF
Rapamycin (nM)
e
Quantification of local sensitivity for CHO-Gal4
0.05 nM Rapa.
y= 0.9576x - 0.9633
50 nM Rapa.
y= 1.8472x - 1.6255
Log output (iRFP)
Log input (EGFP)
f
U2OS-1TetO
0 nM
0.1 nM
0.5 nM
1 nM
n=1.16
n=1.6
n=1.75
n=2
3 nM
5 nM
10 nM
50 nM
n=2.16
n=2.1
n=2.21
n=2.33
Output (iRFP, a.u.)
Input (EGFP, a.u.)
Density
g
U2OS-1TetO
Kd (a.u.)
Rapamycin (nM)
h
U2OS-1TetO
Hill coefficient
Rapamycin (nM)
i
U2OS-1TetO
Bimodality index
Target
TF
Rapamycin (nM)

Supplementary Figure 6: Additional characterizations of the roles of TF clustering in two additional systems.

a-e, Characterizations of the CHO-Gal4 system analogous to the U2OS-7TetO system, including gene regulation function (**a**), dissociation constant (**b**), Hill coefficient (**c**), bimodality index (**d**), and local sensitivity (**e**). Note that a gradient of doxycycline concentrations was used for generating a large range of TF expression levels for CHO-Gal4 system (Methods). Some of the plots in **a** were shown in Fig. 4d. n = 2 biological replicates. **f-i**, Characterizations of the U2OS-1TetO system, including gene regulation function (**f**), dissociation constant (**g**), Hill coefficient (**h**), and bimodality index (**i**). Note that some of the plots in **f** were shown in Fig. 4e. n = 2 biological replicates. Source data are provided.

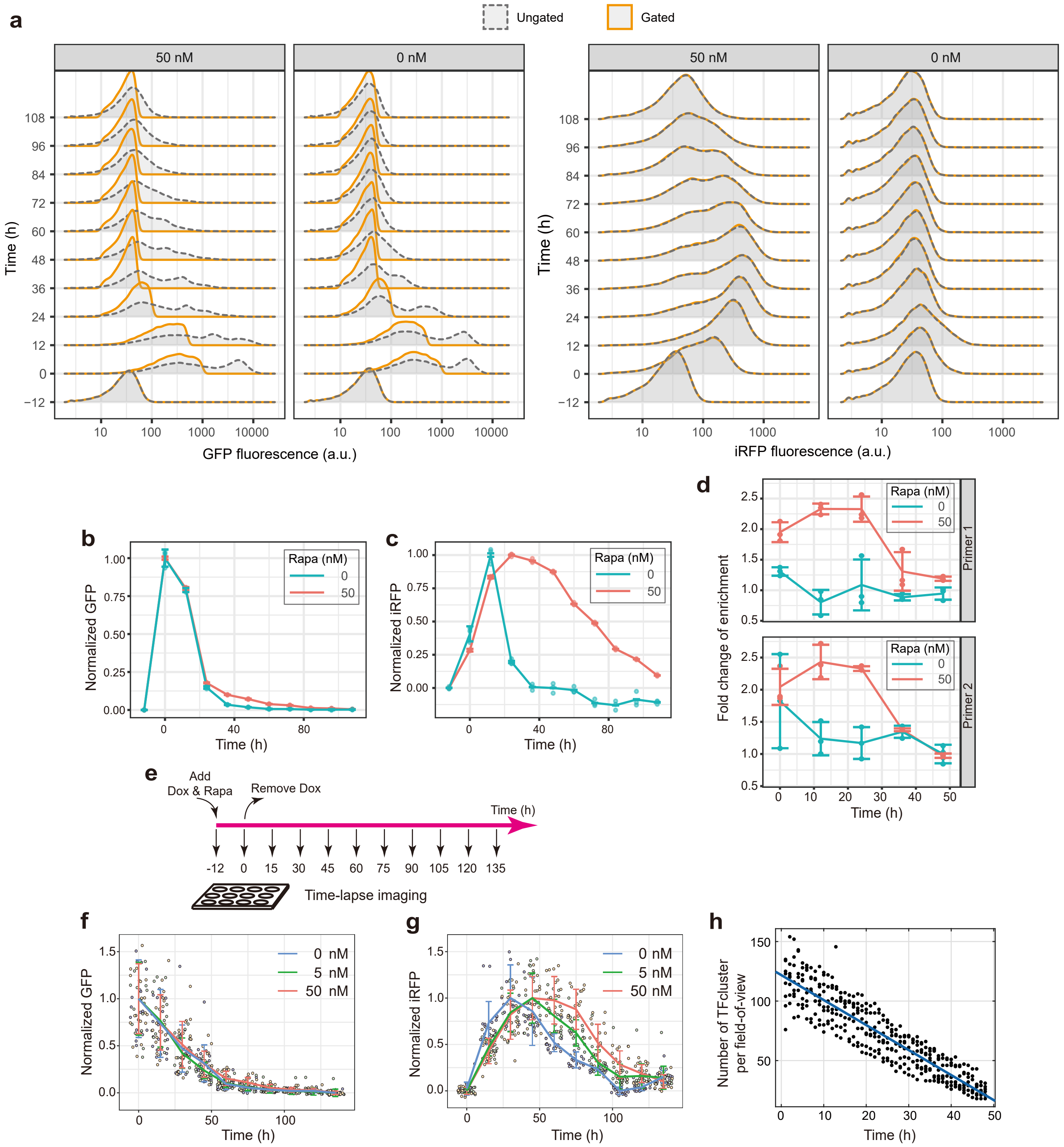

Supplementary Figure 7: Additional characterizations of sustained transcription responses conferred by TF clustering.

a, Gated and ungated flow cytometry data from the experimental time course illustrated in Fig. 5b. Note that gated and ungated iRFP distributions looked similar. **b-c**, Analogous plots to Fig. 5c-f using ungated flow cytometry data. Data are presented as mean ± S.D. (n = 3 biological replicates). **d**, RT-qPCR quantification of the reporter gene using two primer sets at indicated time points during the above time course. Data are presented as mean ± S.D. (n = 3 biological replicates). **e-h**, Time-lapse imaging data further support sustained transcription responses conferred by TF clustering. CHO-Gal4 cells were subjected to a 12 hr pulse of doxycycline (0.1 μg/mL) treatment and fluorescence images of EGFP and iRFP were taken every hour. Processed data from indicated time points are shown (**e**). See Methods for details regarding image processing. Data were normalized in order to compare time scales. Results from three rapamycin conditions show that, while TF concentrations decayed similarly (**f**), the iRFP levels displayed more sustained activation for conditions with higher rapamycin concentrations (**g**). Each point indicates result from one field-of-view. Data are presented as mean ± S.D. n = 19, 20, 20 field-of-views for 0, 5, and 50 nM rapamycin, respectively. During the time course, the number of TF clusters per field-of-view decreased gradually (**h**, n = 8 filed-of-views). Source data are provided.

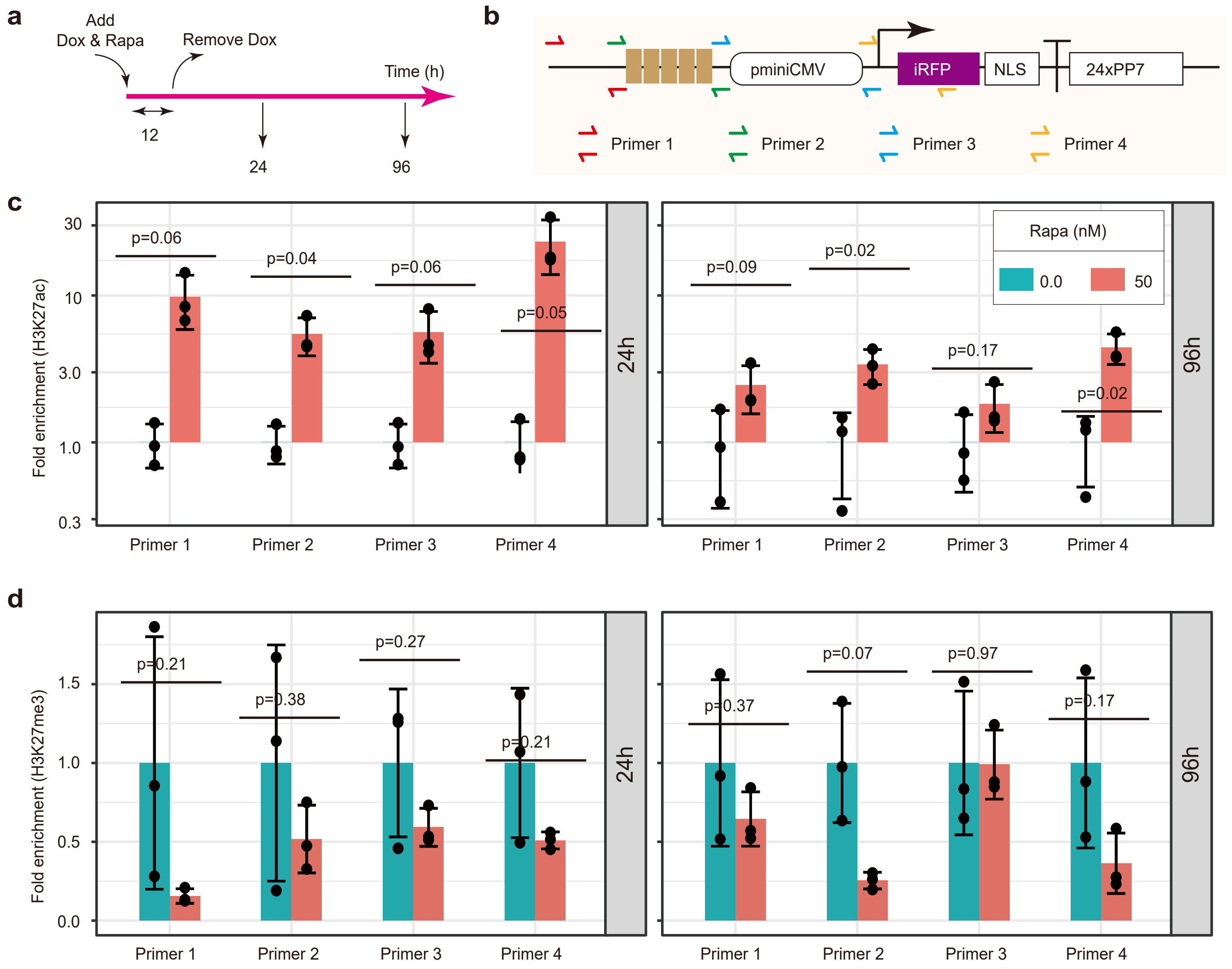

Supplementary Figure 8: Quantifications of the reporter genes' epigenetic states after transient signal by ChIP-qPCR.

a-b, Experiment design. CHO-Gal4 cells were subjected to a 12 hr pulse of doxycycline (0.1 µg/mL) treatment and ChIP-qPCR quantifications were carried out at indicated time points (**a**) with four separate primer sets (**b**). Bglap2 and Cdx2 were used as reference genes. **c-d**, Fold-enrichments of H3K27ac (**c**) and H3K27me3 (**d**) for different primer sets at different conditions and time points. Enrichment scores were normalized by the condition without rapamycin at each condition for each primer set. Data are presented as mean ± S.D. (n = 3 biological replicates). p values were calculated by two-sided t-test. Source data are provided, which contain details of statistical tests.

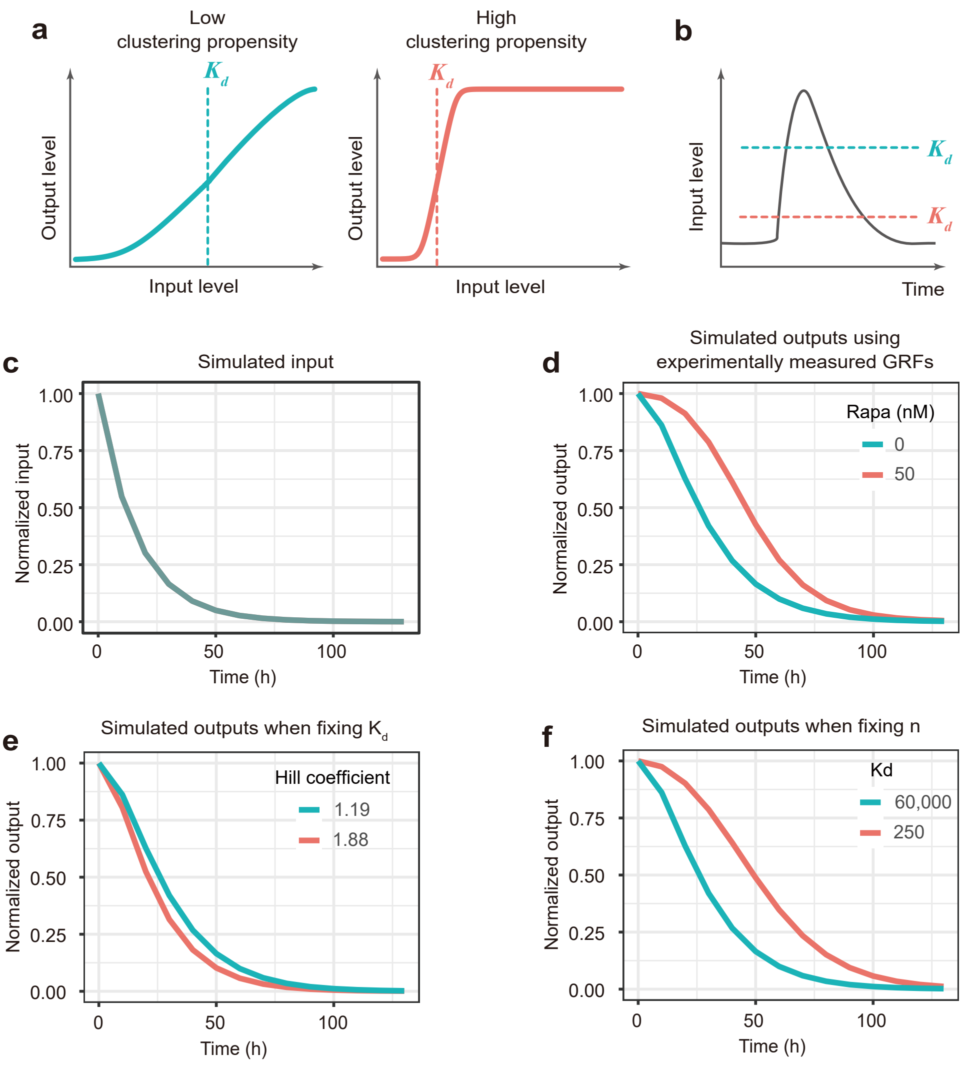

Supplementary Figure 9: A potential mechanism for the sustained transcriptional response conferred by TF clustering.

a-b, Schematic for the potential mechanism underlying sustained transcriptional response conferred by TF clustering. As we have demonstrated, TF clustering results in an gene regulation function with decreased dissociation constant K_d and increased ultrasensitivity (**a**). The different K_d values lead to different thresholds of the TF level for the activation (and deactivation) of the target gene, resulting in a longer duration of activation at high TF clustering propensity (**b**). **c-d**, Simulations with experimental gene regulation functions recapitulated the sustained transcriptional activation. Two populations of cells (5000 cells each) with the same initial input (TF) level distribution at t =0 were simulated for the indicated time course. The production rates of the target protein were calculated by the experimentally measured gene regulation function at zero or 50 nM rapamycin (Supplementary Fig. 6a-c). The half-life for both the TF and the target protein was estimated to be ~12 hours based on Fig. 5e. Normalized population-averaged input (**c**) and output (**d**) signals are shown. **e-f**, Simulated outputs when assuming that TF clustering only modulated ultrasensitivity but not effective binding affinity (**e**) or that TF clustering only modulated effective binding affinity but not ultrasensitivity (**f**). Red lines are outputs from the assumed conditions.

Supplementary Table 1. List of commercial reagents used in the study.

Reagent	Manufacturer	Catalog number
HindIII-HF	NEB	R3104S
EcoRI-HF	NEB	R3101V
NheI-HF	NEB	R3131S
Age I	NEB	R0552S
BamHI-HF®	NEB	R3136S
XbaI	NEB	R0145S
NotI-HF	NEB	R3189S
SpeI-HF	NEB	R3133S
AflII	NEB	R0520S
BstBI	NEB	R0519S
PrimeSTAR Max DNA Polymerase	TAKARA	R045B
DH5α	CWBIO	CW0808S
HiPure Plasmid Kits	Magen	P1001-02
Dulbecco’s Modified Eagle Medium	Gibco	C11995500CP
Fetal bovine serum (FBS)	Gibco	10099141
RPMI 1640 media	Gibco	C11875500CP
BASIC DPBS, no Calcium, no Magnesium	Gibco	C14190500CP
Pen Strep Glutamine (100X)	Gibco	10378-016
Lipofectamine®LTX & PLUS™	Thermo	15338100
Hygromycin	Roche	10843555001
Puromycin Dihydrochloride	Life	A1113803
Doxycycline	Clotech	631311
Rapamycin	Harvey	R31345
Fluoro Brite DMEM	Gibco	A1896701
HiPure Gel Pure DNA Micro Kit	Magen	D2110
RaPure Total RNA Micro Kit	Magen	R4012-02
iScript™ cDNA Synthesis Kit	BIORAD	#1708891
GOTAQ qPCR Master Mix	Promega	A6001
SimpleChIP Kit	Cell Signaling	#CST 9003S
Tri-Methyl-Histone H3 (Lys27) (C36B11) Rabbit mAb	CST	9733S
Acetyl-Histone H3 (Lys27) (D5E4) XP Rabbit mAb	CST	8173S
Rnase/Dnase free water	TIANGEN	RT121-02
RNase and DNase Away	Beyotime Biotech	R0123
Deoxynucleotide (dNTP) Solution Set	Sangon Biotech	A500058-0001
Aminoallyl-dUTP-Cy5	Jena Bioscience	NU-803-CY5-S
DNase I	NEB	M0303S
E. coli DNA Pol I	NEB	M0209S
0.5 M EDTA	Beyotime Biotech	ST066
