# Supplementary material for: Modulating gene regulation function by chemically controlled transcription factor clustering
Source: Nat Commun. 2022 May 13;13:2663. doi: 10.1038/s41467-022-30397-2 (PMC9106659; doi:10.1038/s41467-022-30397-2)
Supplement: Supplementary file 2 — Reporting Summary [file 41467_2022_30397_MOESM2_ESM.pdf]

nature portfolio

Corresponding author(s): Yihan Lin

Last updated by author(s): 04/20/2022

Reporting Summary

Nature Portfolio wishes to improve the reproducibility of the work that we publish. This form provides structure for consistency and transparency in reporting. For further information on Nature Portfolio policies, see our Editorial Policies and the Editorial Policy Checklist.

Statistics

For all statistical analyses, confirm that the following items are present in the figure legend, table legend, main text, or Methods section.

n/a	Confirmed	
☐	☒	The exact sample size (*n*) for each experimental group/condition, given as a discrete number and unit of measurement
☐	☒	A statement on whether measurements were taken from distinct samples or whether the same sample was measured repeatedly
☐	☒	The statistical test(s) used AND whether they are one- or two-sided<br>*Only common tests should be described solely by name; describe more complex techniques in the Methods section.*
☒	☐	A description of all covariates tested
☒	☐	A description of any assumptions or corrections, such as tests of normality and adjustment for multiple comparisons
☐	☒	A full description of the statistical parameters including central tendency (e.g. means) or other basic estimates (e.g. regression coefficient) AND variation (e.g. standard deviation) or associated estimates of uncertainty (e.g. confidence intervals)
☐	☒	For null hypothesis testing, the test statistic (e.g. *F*, *t*, *r*) with confidence intervals, effect sizes, degrees of freedom and *P* value noted<br>*Give P values as exact values whenever suitable.*
☒	☐	For Bayesian analysis, information on the choice of priors and Markov chain Monte Carlo settings
☒	☐	For hierarchical and complex designs, identification of the appropriate level for tests and full reporting of outcomes
☒	☐	Estimates of effect sizes (e.g. Cohen's *d*, Pearson's *r*), indicating how they were calculated

Our web collection on statistics for biologists contains articles on many of the points above.

Software and code

Policy information about availability of computer code

Data collection	Fusion 2.3.0.36 (Andor, Oxford Instruments) was used to collect all confocal image; BD FACSDiva Software version 8.0.2 (BD Biosciences) was used to collect all the Flow Cyto. data.
Data analysis	Public and commercial software: Matlab R2017b (MathWorks); EasyFlow (https://antebilab.github.io/easyflow); R version 4.0.2 (The R Foundation for Statistical Computing); ImageJ 1.53f51 with Fiji package (https://imagej.net/software/fiji/)<br>Custom codes (codes are available from the corresponding author on request): Custom Matlab codes were used to automatically segment individual cells and to identify TF clusters within each cell; A custom Matlab program with graphical user interface was used for semi-automatic identification and tracking of each nascent transcription site throughout the time series; An R code was used in this study to simulate the memory-like behavior of TF with ultrasensitive input-output function.

For manuscripts utilizing custom algorithms or software that are central to the research but not yet described in published literature, software must be made available to editors and reviewers. We strongly encourage code deposition in a community repository (e.g. GitHub). See the Nature Portfolio guidelines for submitting code & software for further information.

Data

Policy information about availability of data

All manuscripts must include a data availability statement. This statement should provide the following information, where applicable:

- Accession codes, unique identifiers, or web links for publicly available datasets
- A description of any restrictions on data availability
- For clinical datasets or third party data, please ensure that the statement adheres to our policy

Source data are provided with this paper for reproduce all the main figures and supplementary figures. All other data are available from the corresponding author on request (Confocal raw data and flow cytometry raw data are too large to upload to web.).

Field-specific reporting

Please select the one below that is the best fit for your research. If you are not sure, read the appropriate sections before making your selection.

☒ Life sciences ☐ Behavioural & social sciences ☐ Ecological, evolutionary & environmental sciences

For a reference copy of the document with all sections, see nature.com/documents/nr-reporting-summary-flat.pdf

Life sciences study design

All studies must disclose on these points even when the disclosure is negative.

Sample size	No sample size calculation was performed.<br>A sample size of >15 cells is regarded as sufficient.<br>For bulk experiments, sample size is determined by the type of experiment, and a sample size >=2 is sufficient.
Data exclusions	No data were excluded from the analyses.
Replication	For experiments with statistical tests, at least three biological replicates were used in this study. For others, experiments were repeated at least twice. All attempts at replication were successful.
Randomization	Randomization is not relevant because of the nature of the measurements. We always set control and experiment group for this study.
Blinding	Blinding is not relevant because of the nature of the measurements. We always set control and experiment group for this study.

Reporting for specific materials, systems and methods

We require information from authors about some types of materials, experimental systems and methods used in many studies. Here, indicate whether each material, system or method listed is relevant to your study. If you are not sure if a list item applies to your research, read the appropriate section before selecting a response.

Materials & experimental systems

n/a	Involved in the study	
☐	☒	Antibodies
☐	☒	Eukaryotic cell lines
☒	☐	Palaeontology and archaeology
☒	☐	Animals and other organisms
☒	☐	Human research participants
☒	☐	Clinical data
☒	☐	Dual use research of concern

Methods

n/a	Involved in the study	
☒	☐	ChIP-seq
☐	☒	Flow cytometry
☒	☐	MRI-based neuroimaging

Antibodies

Antibodies used	The following antibodies were used in this study for CHIP-qPCR experiments: Tri-Methyl-Histone H3 (Lys27) (C36B11) Rabbit mAb (CST #9733S, 1:100 dilution), Acetyl-Histone H3 (Lys27) (D5E4) XP® Rabbit mAb (CST #8173, 1:100 dilution).
Validation	Those two antibodies can be used in Chromatin Immunoprecipitation experiment. They have reactivity in human, mouse, rat and monkey species, as validated by the manufacturer.

Eukaryotic cell lines

Policy information about cell lines

Cell line source(s)	U2OS cells and CHO cells are from ATCC.
Authentication	The cell lines were authenticated by comparing the cell morphology and doubling time with data from the provider.
Mycoplasma contamination	We tested mycoplasma contamination of all cell lines in this study, and the results were negative.
Commonly misidentified lines (See ICLAC register)	We affirm that there are no commonly misidentified lines.

Flow Cytometry

Plots

Confirm that:

☒ The axis labels state the marker and fluorochrome used (e.g. CD4-FITC).

☒ The axis scales are clearly visible. Include numbers along axes only for bottom left plot of group (a 'group' is an analysis of identical markers).

☒ All plots are contour plots with outliers or pseudocolor plots.

☒ A numerical value for number of cells or percentage (with statistics) is provided.

Methodology

Sample preparation	All flow cytometry assays in this study were based on cell lines. Cells were cultured in plastic-bottom wells and dispersed by trypsin (3 to 4 min for all cell lines). Signal collection was completed in 30 minutes after dispersion. No tissue processing steps were used in this study.
Instrument	Fluorescence signals of all samples were collected on a BD Fortessa SORP flow cytometer.
Software	A matlab software, i.e., easyflow (from the Antebi Lab at Weizmann Institute of Science, https://antebilab.github.io/easyflow), was used in this study to analyze the fluorescence signals.
Cell population abundance	In each flow cytometry assays in this study, at least 10,000 cells were collected.
Gating strategy	For all flow cytometry data, because we are working with cell lines, we only gated for singlets based on FSC/SCC signals. No other gating was applied except for Fig. 5. In Fig. 5c-f, gates were chosen based on the level of EGFP signal at each time point (1~100 at -12 h, 10~1000 at 0 h, 10~500 at 12 h, 10~100 at 24 h and 10~50 at other time points). Ungated data was also shown. A figure exemplifying the gating strategy is shown in Supplementary 1g.

☒ Tick this box to confirm that a figure exemplifying the gating strategy is provided in the Supplementary Information.
