# Supplementary material for: Modulating gene regulation function by chemically controlled transcription factor clustering
Source: Nat Commun. 2022 May 13;13:2663. doi: 10.1038/s41467-022-30397-2 (PMC9106659; doi:10.1038/s41467-022-30397-2)
Supplement: Supplementary file 4 — Description of Additional Supplementary Files [file 41467_2022_30397_MOESM4_ESM.pdf]

Title: Supplementary Movie 1:

Description: Two-color time-lapse movie showing the spatiotemporal interactions between TF clusters and nascent transcription sites. Time-lapse movie of the EGFP fluorescence (TF clusters) and mCherry fluorescence (PCP-3xmCherry) of a single cell under the condition as in Fig. 3d. The frame rate is 1 frame per 5 min and the total movie time is 355 min.

Title: Supplementary Movie 2:

Description: Transcriptional bursting dynamics under different rapamycin conditions. Time-lapse movies of the mCherry fluorescence (PCP-3xmCherry) for four representative single cells under indicated rapamycin conditions are shown. The frame rate is 1 frame per 10 min and the total movie time is 500 min. These movies are related to Supplementary Fig. 4a.
